# Interactive effects of Composite Dietary Antioxidant Index with Body Mass Index for the risk of stroke among U.S. adults: insight from NHANES 2001–2018

**DOI:** 10.3389/fnut.2024.1378479

**Published:** 2024-06-07

**Authors:** Xi Li, Xin Hu, Chao You

**Affiliations:** ^1^West China School of Medicine, West China Hospital, Sichuan University, Chengdu, China; ^2^Department of Clinical Research Management, West China Hospital, Sichuan University, Chengdu, Sichuan, China; ^3^Department of Neurosurgery, West China Hospital, Sichuan University, Chengdu, Sichuan, China

**Keywords:** Composite Dietary Antioxidant Index CDAI, Body Mass Index (BMI), stroke, NHANES (National Health and Nutrition Examination Survey), adults

## Abstract

**Background:**

This cross-sectional study aims to explore the interactive effects of the Composite Dietary Antioxidant Index (CDAI) and Body Mass Index (BMI) on stroke risk among U.S. adults, utilizing data from the National Health and Nutrition Examination Survey (NHANES) conducted between 2001 and 2018.

**Methods:**

The analysis involved 42,042 participants from a representative sample of non-institutionalized U.S. civilians, selected through a stratified, multistage probability sampling method. Dietary intake data were collected over two 24-h periods using the Automated Multiple-Pass Method. The study calculated a modified CDAI to assess dietary antioxidant intake, excluding supplements and water sources. Statistical methods included multivariable logistic regression and Generalized Additive Models (GAM) to evaluate the interaction between CDAI scores and BMI in relation to stroke risk, adjusting for a wide range of demographic, lifestyle, and health covariates.

**Results:**

The research identified a significant interaction between CDAI scores and BMI categories in stroke risk assessment. While a negative correlation was observed between CDAI scores and stroke risk across the total population (OR 0.97, 95% CI 0.96–0.99), this relationship varied notably across different BMI groups. In participants with a BMI ≥25, a statistically significant negative association persisted, displaying a non-linear pattern. The study also revealed an inflection point in the CDAI score, indicating a shift in the relationship between dietary antioxidants and stroke risk.

**Conclusion:**

This study underscores the complex interaction between dietary antioxidant intake and BMI in determining stroke risk among U.S. adults. The findings suggest that individuals with higher BMI may experience more pronounced benefits from dietary antioxidants in stroke prevention. These insights could inform targeted dietary recommendations and public health strategies aimed at reducing stroke risk, particularly in populations with higher BMI. Further research is needed to fully understand these interactions and their implications for stroke prevention guidelines.

## 1 Introduction

Stroke, with its high morbidity, mortality, and resultant heavy socio-economic burden, is a pressing global health issue. The 2019 Global Burden of Disease data highlights this severity, showing a substantial increase in stroke-related statistics from 1990 to 2019: incident strokes rose by 70%, prevalent strokes by 85%, stroke-related deaths by 43%, and disability-adjusted life years (DALYs) due to stroke by 32% (1). In the United States, between 1999 and 2018, the crude and age-standardized prevalence rates of stroke were 2.84 and 3.10%, respectively, impacting an estimated 7.3 million individuals (2). These figures underscore the importance of identifying populations at high risk of stroke for primary prevention.

In the context of cardiovascular diseases (CVD), including stroke, dietary factors are pivotal. Chronic inflammation, marked by the continuous presence of pro-inflammatory cytokines in the bloodstream, significantly contributes to CVD. This inflammation, often a result of tissue injury and the release of cytokines like TNF-α, interleukin-1, and IL-6, is closely associated with dietary habits (3). The Composite Dietary Antioxidant Index (CDAI) is a tool that integrates multiple dietary antioxidants, such as carotenoids, selenium, vitamin A, vitamin C, vitamin E, and zinc, providing a comprehensive score reflecting an individual’s antioxidant profile (4). The formulation of the CDAI is based on the understanding that these antioxidants collectively aid in combating oxidative stress and inflammation, which are critical in the development and progression of CVD (5, 6). Thus, the CDAI is not just a measure of dietary impact on health but also a potential predictor or mitigator of stroke risk.

The interplay between dietary antioxidants and stroke has been a focal point of recent research, revealing a complex relationship. Nevertheless, findings across studies have been inconsistent. Two cohort studies by Rautiainen et al. and Colarusso et al. (7, 8) which measured Total Antioxidant Capacity (TAC) and Nonenzymatic Antioxidant Capacity (NEAC), respectively, both reported a protective effect of dietary antioxidants against stroke. Conversely, a study by Hantikainen et al. (9) which also used NEAC to assess dietary antioxidant intake, found no link between a high-antioxidant diet and stroke risk. At the same time, a number of recent studies have further analyzed the association between CDAI and stroke by using CDAI as a measure of dietary antioxidant intake, and found that there is a nonlinear association between CDAI and stroke (10–13).

Moreover, a high Body Mass Index (BMI) has been recognized as a significant risk factor for stroke, underscoring the need for careful monitoring of BMI (14–16). Pro-inflammatory cytokines not only contribute to CVD but also mediate obesity-related conditions such as insulin resistance and hypertension (17). The relationship between weight gain and cardiovascular health may be mediated through inflammatory pathways, with studies indicating that obesity’s link to CVD is partly facilitated by alterations in inflammatory mediators like cytokines and chemokines (18, 19). Although the preventive role of dietary indicators in obese individuals is established, there remains a gap in research specifically examining the interaction between diet and BMI on stroke risk. Therefore, this study aims to explore the interaction between CDAI and BMI on stroke risk in US adults and further evaluate the existence of possible effect modifiers.

## 2 Materials and methods

### 2.1 Study population

**Figure 1:**
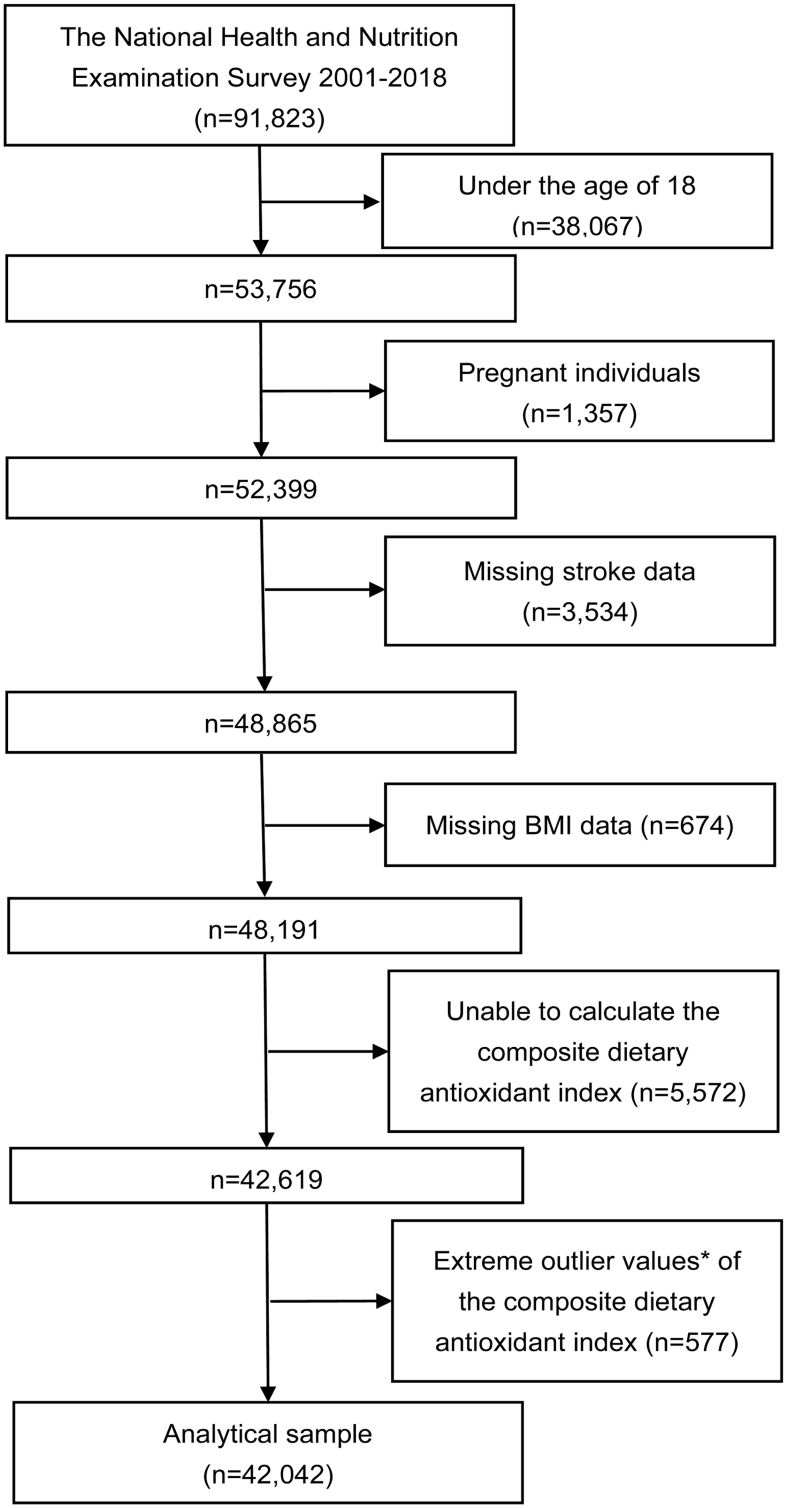
Flowchart of the study. ^*^Extreme outlier values, defined as those over 3 standard deviations from the mean.

The NHANES is an extensive cross-sectional study that focuses on a representative group of non-institutionalized civilians in the U.S. This survey utilized a stratified, multistage probability approach for sampling. Participants provided detailed accounts of their dietary consumption over two successive 24-h periods. The initial data collection occurred face-to-face at a mobile examination center, followed by a telephone interview conducted 3–10 days afterwards. Rigorous interviewer training lasting 1 week and standardized measurement tools (such as cups and spoons) were implemented to enhance data precision. Dietary recall was conducted using the U.S. Department of Agriculture’s Automated Multiple-Pass Method. Ethical approval for NHANES was granted by the National Center for Health Statistics’ Research Ethics Review Committee, with informed consent obtained from all respondents. The current study analyzed data spanning from 2001 to 2018, encompassing 91,823 participants. Exclusion criteria included individuals under 18 years (38,067 individuals), pregnant women (1,357 individuals), missing stroke information (3,534 individuals), missing BMI data (674 individuals), those for whom the CDAI could not be computed (5,572 individuals), and extreme CDAI values (± 3 standard deviations) (577 individuals), leading to the inclusion of 42,042 participants for analysis (Figure 1).

### 2.2 Calculation of CDAI

Data on dietary antioxidant intake and other food components were collected through 24-h dietary recall interviews. Participants recounted specific foods and drinks consumed in the 24-h period preceding the interview. To quantify dietary antioxidant exposure, we adopted a modified CDAI as developed by Wright et al. (4, 20) This involved standardizing the intake of six antioxidants (carotenoids, selenium, vitamin A, vitamin C, vitamin E, zinc) by calculating the deviation of individual intake from the mean and summing these standardized values to represent the CDAI. Notably, antioxidants from supplements, medications, or plain water were excluded from this calculation. The formula was shown as following:


$$\mathrm{CDAI}=\overset{6}{\underset{i=1}{\sum }}\frac{\mathrm{Individual}\;\mathrm{Intake}-\mathrm{Mean}}{SD}$$

### 2.3 Definition of stroke

Stroke identification relied on self-reported data from the Medical Condition Questionnaire. Participants indicated whether they had been diagnosed with a stroke by a health professional, thus classifying them into stroke and non-stroke categories. The NHANES database used self-report questionnaires to collect stroke data, limiting the ability to distinguish between ischemic and hemorrhagic strokes. Despite this limitation, self-reported stroke data have been shown to be relatively accurate in the U.S. population and are consistent with methodologies in previous epidemiological studies using NHANES data (21, 22).

### 2.4 Covariates

To reduce variability, the study included a diverse range of covariates. These encompassed demographic details such as age, gender, level of education, the poverty income ratio (PIR), and race/ethnicity; lifestyle attributes like physical activity, smoking, and drinking habits; data from physical examinations including blood pressure and BMI; and self-reported health information, covering medical and drug histories. BMI was derived from height and weight measurements. The PIR was calculated by dividing the family income by the poverty threshold and was classified into three categories: low (<1.3), medium (1.3–3.5), and high (>3.5). Smoking status was categorized as never smokers (those who have smoked less than 100 cigarettes in their lifetime), former smokers (those who have smoked 100+ cigarettes in their lifetime but had quit by the time of the survey), and current smokers (those who have smoked 100+ cigarettes in their lifetime and continue to smoke at least every few days). Current drinking included heavy (≥3 drinks per day for women; ≥4 drinks per day for men; ≥5 binge drinking days per month), moderate (≥2 drinks per day for women; ≥3 drinks per day for men; ≥2 binge drinking days per month), and mild (other). Physical activity was quantified in the metabolic equivalent of task per week (METs/week), divided into low (<600 METs/week), moderate (600–1,199 METs/week), and high (≥1,200 METs/week) activity levels. The estimated glomerular filtration rate (eGFR) was calculated using the 2009 Chronic Kidney Disease Epidemiology Collaboration (CKD-EPI) equation based on serum creatinine (23). A diagnosis of hypertension is established when any of the following criteria are met: a blood pressure reading of 140/90 mmHg or higher, a clinical diagnosis of hypertension, or the utilization of antihypertensive medication. Diabetes was confirmed based on any of the following: doctor diagnosis, glycohemoglobin (HbA1c) >6.5%, fasting blood glucose ≥7.0 mmol/L, random blood glucose ≥11.1 mmol/L, or a two-hour glucose ≥11.1 mmol/L in an oral glucose tolerance test (OGTT). Hyperlipidemia is diagnosed in the presence of either hypertriglyceridemia (triglycerides [TG] at or above 150 mg/dL), hypercholesterolemia (total cholesterol [TC] at or above 200 mg/dL, low-density lipoprotein cholesterol at or above 130 mg/dL, or high-density lipoprotein cholesterol below 40 mg/dL in men and below 50 mg/dL in women), or the use of lipid-lowering medications. Sleep-related factors were assessed using “trouble sleeping” as the criterion, specifically based on respondents’ answers to the question “Have you ever told a doctor or other health professional that you have trouble sleeping?” In the NHANES.

### 2.5 Statistical analysis

This study is a cross-sectional analytical study. Our analysis began with a comparison of the initial data. Based on BMI, participants were divided into two groups according to the presence of stroke for baseline characteristic analysis. We assessed the impact of various components of CDAI on inflammation, acknowledging that these components might influence outcomes differently, potentially showing exponential increases or decreases. The distribution of CDAI components across groups was detailed using median values and interquartile ranges. For continuous data, we presented means with standard errors, and for categorical data, we used percentage representations. We applied Student’s t-test or the chi-squared test for normally distributed variables, while skewed variables were analyzed using non-parametric methods or Fisher’s exact probability test.

In our comprehensive analysis, which included the total population and subgroups stratified by BMI, we employed a multivariable logistic regression model to calculate odds ratios (ORs) and 95% confidence intervals (CIs), incorporating the CDAI score as an independent variable in both continuous form and by quartiles (Q1 (−7.18 to −2.26, *n* = 10,391), Q2 (−2.26 to −0.31, *n* = 10,497), Q3 (−0.31 to 2.15, *n* = 10,599), Q4 (2.15 to 12.80, *n* = 10,555)) to investigate associations with stroke risk. We conducted linear trend tests to evaluate linear relationships and utilized a Generalized Additive Model (GAM) with smoothed curve fitting to probe non-linear associations. For non-linear relationships, we applied a segmented logistic regression model, analyzing data on either side of a determined inflection point separately. The optimal model for examining the relationship between CDAI scores and stroke was identified through a log-likelihood ratio test.

Subgroup analyses and interaction tests were carried out considering factors such as sex, age, race/ethnicity, smoking status, alcohol consumption, hypertension, diabetes, and hyperlipidemia, with adjustments for various covariates. Additionally, to assess the impact of sleep-related factors in this study, “trouble sleeping” was included as one of the additional covariates in a sensitivity analysis. As the data on “trouble sleeping” was only available for the period from 2008 to 2018, a narrower dataset was utilized for the analysis. Our statistical analyses were performed using R software (version 4.2.0) and EmpowerStats, with statistical significance set at a *p*-value below 0.05.

## 3 Results

### 3.1 Baseline characteristics

Table 1 outlines the baseline characteristics segmented by BMI within stroke and non-stroke cohorts. Among participants, 12,223 fell into the category with a BMI under 25 kg/m^2^, while 29,819 were classified into the category with a BMI of 25 kg/m^2^ or above. In the lower BMI group, no significant differences were noted in sex (*p* = 0.221) or diastolic blood pressure (DBP) (*p* = 0.919) between those with and without stroke. However, significant distinctions were observed in age, educational level, PIR, racial/ethnic identity, smoking status, alcohol use, MET levels, BMI, systolic blood pressure (SBP), eGFR, and the presence of diabetes, hypertension, and hyperlipidemia. The data suggested that, for both men and women, the likelihood of stroke increased with age, lower education and economic status, being non-Hispanic white or black, smoking history, prior alcohol use, reduced physical activity, elevated BMI and SBP, decreased eGFR, and the presence of diabetes, hypertension, and hyperlipidemia. Furthermore, all variables except sex showed significant differences between the stroke and non-stroke groups in the BMI ≥25 kg/m^2^ category.

**Table 1:** Baseline characteristics of subjects.

Characteristics	BMI < 25 kg/m^2^				BMI ≥ 25 kg/m^2^			
	Total *n* = 12,223	Non-stroke *n* = 11,827	Stroke *n* = 396	*p*-value	Total *n* = 29,819	Non-stroke *n* = 28,575	Stroke *n* = 1,244	*p*-value
Age (years)	47.34 ± 19.42	46.66 ± 19.20	67.83 ± 14.05	<0.001	51.04 ± 17.15	50.42 ± 17.03	65.33 ± 13.19	<0.001
Sex, *n* (%)				0.221				0.498
Male	5,773 (47.23%)	5,574 (47.13%)	199 (50.25%)		15,070 (50.54%)	14,453 (50.58%)	617 (49.60%)	
Female	6,450 (52.77%)	6,253 (52.87%)	197 (49.75%)		14,749 (49.46%)	14,122 (49.42%)	627 (50.40%)	
Education level, *n* (%)				<0.001				<0.001
Less than high school	2,808 (23.00%)	2,649 (22.43%)	159 (40.25%)		7,921 (26.58%)	7,486 (26.22%)	435 (35.00%)	
High school	2,713 (22.22%)	2,629 (22.26%)	84 (21.27%)		7,126 (23.92%)	6,778 (23.74%)	348 (28.00%)	
More than high school	6,686 (54.77%)	6,534 (55.32%)	152 (38.48%)		14,749 (49.50%)	14,289 (50.04%)	460 (37.01%)	
PIR, *n* (%)				<0.001				<0.001
Low	3,359 (29.77%)	3,210 (29.42%)	149 (40.05%)		8,339 (30.43%)	7,887 (30.03%)	452 (39.65%)	
Medium	4,232 (37.51%)	4,079 (37.39%)	153 (41.13%)		10,674 (38.95%)	10,187 (38.78%)	487 (42.72%)	
High	3,691 (32.72%)	3,621 (33.19%)	70 (18.82%)		8,393 (30.62%)	8,192 (31.19%)	201 (17.63%)	
Race/ethnicity, *n* (%)				<0.001				<0.001
Non-Hispanic White	5,937 (48.57%)	5,723 (48.39%)	214 (54.04%)		12,925 (43.34%)	12,303 (43.06%)	622 (50.00%)	
Non-Hispanic Black	2,168 (17.74%)	2079 (17.58%)	89 (22.47%)		6,726 (22.56%)	6,376 (22.31%)	350 (28.14%)	
Mexican American	1,408 (11.52%)	1,373 (11.61%)	35 (8.84%)		5,530 (18.55%)	5,396 (18.88%)	134 (10.77%)	
Others	2,710 (22.17%)	2,652 (22.42%)	58 (14.65%)		4,638 (15.55%)	4,500 (15.75%)	138 (11.09%)	
Smoking, *n* (%)				<0.001				<0.001
Never	6,554 (53.62%)	6,414 (54.23%)	140 (35.35%)		16,131 (54.10%)	15,631 (54.70%)	500 (40.19%)	
Former	2,409 (19.71%)	2,280 (19.28%)	129 (32.58%)		8,022 (26.90%)	7,542 (26.39%)	480 (38.59%)	
Now	3,254 (26.62%)	3,127 (26.44%)	127 (32.07%)		5,649 (18.94%)	5,385 (18.85%)	264 (21.22%)	
Not reported	6 (0.05%)	6 (0.05%)	0 (0.00%)		17 (0.06%)	17 (0.06%)	0 (0.00%)	
Drinking, *n* (%)				<0.001				<0.001
Never	1,582 (12.94%)	1,518 (12.84%)	64 (16.16%)		3,900 (13.08%)	3,719 (13.01%)	181 (14.55%)	
Former	1,629 (13.33%)	1,512 (12.78%)	117 (29.55%)		5,109 (17.13%)	4,695 (16.43%)	414 (33.28%)	
Mild	3,865 (31.62%)	3,760 (31.79%)	105 (26.52%)		9,116 (30.57%)	8,801 (30.80%)	315 (25.32%)	
Moderate	1858 (15.20%)	1827 (15.45%)	31 (7.83%)		3,997 (13.40%)	3,899 (13.64%)	98 (7.88%)	
Heavy	2,306 (18.87%)	2,273 (19.22%)	33 (8.33%)		5,429 (18.21%)	5,309 (18.58%)	120 (9.65%)	
Not reported	983 (8.04%)	937 (7.92%)	46 (11.62%)		2,268 (7.61%)	2,152 (7.53%)	116 (9.32%)	
METs/week, *n* (%)				<0.001				<0.001
Low	2,871 (23.49%)	2,794 (23.62%)	77 (19.44%)		6,745 (22.62%)	6,520 (22.82%)	225 (18.09%)	
Moderate	272 (2.23%)	267 (2.26%)	5 (1.26%)		584 (1.96%)	574 (2.01%)	10 (0.80%)	
Vigorous	6,260 (51.21%)	6,121 (51.75%)	139 (35.10%)		14,083 (47.23%)	13,680 (47.87%)	403 (32.40%)	
Not reported	2,820 (23.07%)	2,645 (22.36%)	175 (44.19%)		8,407 (28.19%)	7,801 (27.30%)	606 (48.71%)	
BMI (kg/m^2^)	22.19 ± 2.01	22.18 ± 2.01	22.33 ± 2.10	0.049	31.88 ± 6.06	31.87 ± 6.07	32.12 ± 5.80	0.008
SBP (mmHg)	121.16 ± 19.92	120.69 ± 19.53	135.44 ± 25.52	<0.001	125.84 ± 18.46	125.50 ± 18.19	133.80 ± 22.44	<0.001
DBP (mmHg)	69.02 ± 11.42	69.02 ± 11.32	68.96 ± 14.12	0.919	71.38 ± 12.17	71.47 ± 12.10	69.34 ± 13.70	<0.001
eGFR (ml/min/1.73 m^2^)	96.07 ± 23.80	96.86 ± 23.29	72.29 ± 26.71	<0.001	91.80 ± 23.62	92.68 ± 23.16	71.35 ± 24.72	<0.001
Diabetes, *n* (%)	1,031 (8.43%)	937 (7.92%)	94 (23.74%)	<0.001	6,472 (21.70%)	5,917 (20.71%)	555 (44.61%)	<0.001
Hypertension, *n* (%)	3,600 (29.47%)	3,305 (27.96%)	295 (74.49%)	<0.001	14,434 (48.42%)	13,399 (46.90%)	1,035 (83.20%)	<0.001
Hyperlipidemia, *n* (%)	6,662 (54.51%)	6,360 (53.78%)	302 (76.26%)	<0.001	22,681 (76.07%)	21,607 (75.62%)	1,074 (86.33%)	<0.001
CDAI	0.40 ± 3.58	0.43 ± 3.59	−0.64 ± 3.22	<0.001	0.19 ± 3.40	0.23 ± 3.40	−0.64 ± 3.33	<0.001

PIR, poverty income ratio; MET, metabolic equivalent of task; BMI, body mass index; SBP, systolic blood pressure; DBP, diastolic blood pressure; eGFR, estimated glomerular filtration rate; CDAI, composite dietary antioxidant index.

**Figure 2:**
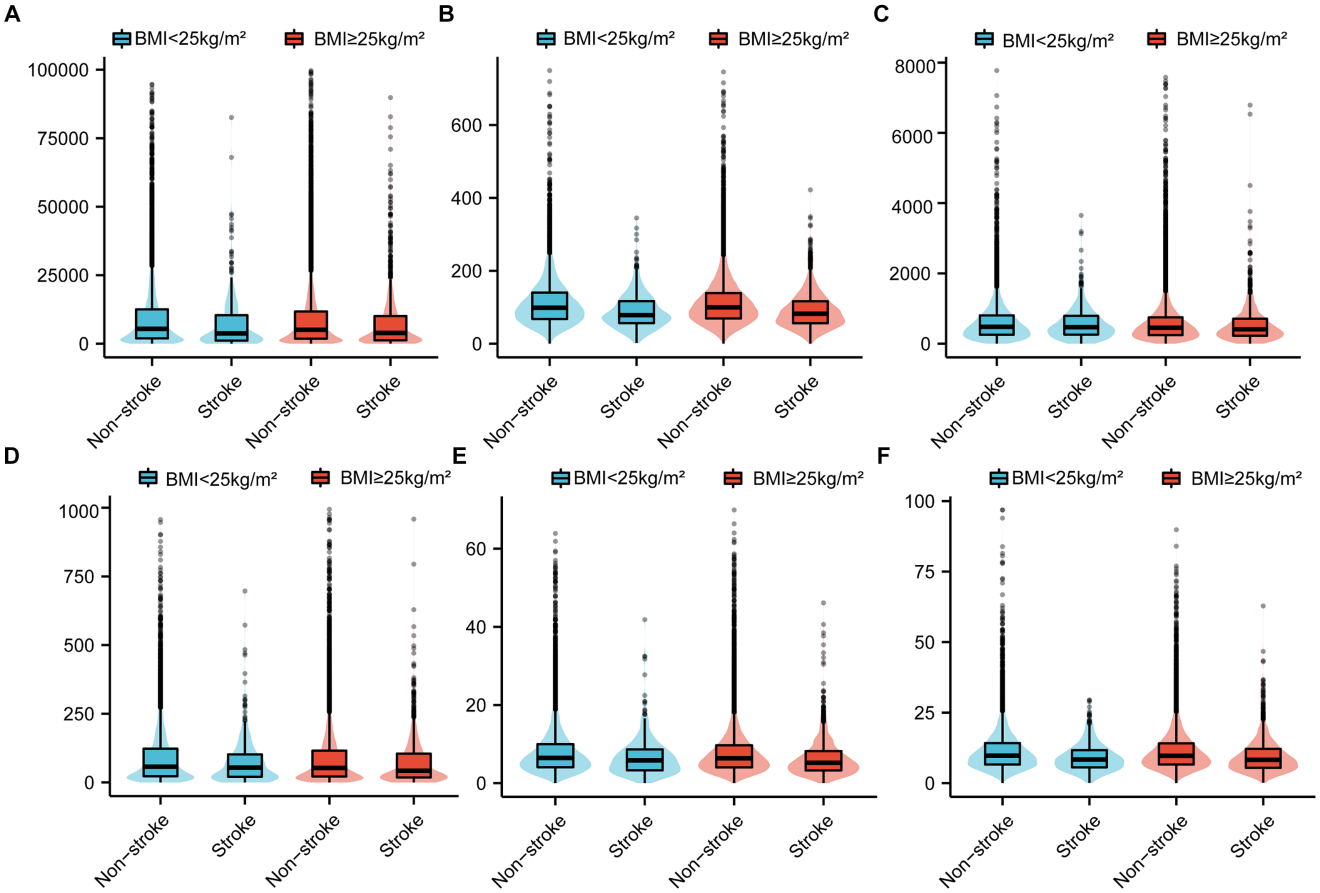
Comparative analysis of CDAI score components [**(A)** Carotenoid; **(B)** Selenium; **(C)** Vitamin A; **(D)** Vitamin C; **(E)** Vitamin E; **(F)** Zinc] between non-stroke and stroke individuals among different BMI groups. CDAI, composite dietary antioxidant index; BMI, body mass index.

Table 2 and Figure 2 present the scores for each CDAI component, categorized by the BMI of individuals in the stroke and non-stroke groups. According to the data in Table 2, vitamin A intake did not show a significant difference between the groups with a BMI under 25 kg/m^2^ (*p* = 0.645). For other CDAI components, significant variances were found between the stroke and non-stroke groups across both BMI categories (less than 25 kg/m^2^ and greater than or equal to 25 kg/m^2^).

**Table 2:** Comparison of each component of CDAI scores between individuals with stroke and individuals without stroke among different BMI groups.

CDAI components	BMI < 25 kg/m^2^				BMI ≥ 25 kg/m^2^			
	Total *n* = 12,223	Non-stroke *n* = 11,827	Stroke *n* = 396	*p*-value	Total *n* = 29,819	Non-stroke *n* = 28,575	Stroke *n* = 1,244	*p*-value
Carotenoid	5263.00 (1855.00–12064.00)	5329.00 (1880.50–12110.00)	3671.50 (1102.25–10035.25)	<0.001	4963.00 (1786.00–11503.50)	5012.00 (1815.50–11576.50)	3888.50 (1214.75–10041.00)	0.007
Selenium	97.10 (66.90–137.60)	97.60 (67.20–138.30)	78.35 (56.30–116.52)	<0.001	98.40 (68.20–136.90)	99.10 (68.90–138.00)	82.10 (56.20–116.10)	<0.001
Vitamin A	472.00 (253.00–786.00)	473.00 (253.00–786.00)	465.00 (257.25–759.25)	0.645	446.00 (243.00–737.00)	448.00 (244.00–738.00)	408.50 (223.50–697.50)	0.002
Vitamin C	55.80 (22.00–118.55)	55.90 (22.10–119.00)	52.65 (20.20–100.15)	0.026	51.60 (21.15–113.40)	51.90 (21.30–113.65)	42.40 (18.00–103.62)	<0.001
Vitamin E	6.34 (4.03–9.67)	6.36 (4.05–9.72)	5.80 (3.27–8.58)	<0.001	6.26 (3.98–9.51)	6.30 (4.01–9.58)	5.21 (3.23–8.16)	<0.001
Zinc	9.58 (6.53–13.82)	9.64 (6.55–13.91)	8.34 (5.57–11.69)	<0.001	9.56 (6.52–13.89)	9.63 (6.56–13.96)	8.22 (5.41–12.12)	<0.001

Data are presented as the median and interquartile ranges (Q1–Q3). CDAI, composite dietary antioxidant index; BMI, body mass index.

### 3.2 Association between CDAI score and stroke risk

Table 3 displays the results of logistic regression analysis, showcasing the association between CDAI scores and the occurrence of stroke. When adjusted for various covariates, including age, sex, education level, PIR, race/ethnicity, smoking, drinking, METs/week, BMI, eGFR, diabetes, hypertension, hyperlipidemia, a negative relationship emerged between the CDAI score and stroke incidence (OR 0.97, 95% CI 0.96–0.99). Further analysis by dividing the CDAI scores into quartiles indicated that participants in the second, third, and fourth quartiles experienced reduced odds of stroke (Q2: OR 0.92, 95% CI 0.80–1.06; Q3: OR 0.80, 95% CI 0.68–0.93; Q4: OR 0.78, 95% CI 0.66–0.92) compared to those in the lowest quartile (Q1). This observation was confirmed by trend tests (*p* < 0.05). However, the relationship between CDAI scores and stroke varied across different BMI categories. In patients with BMI <25, the negative association between CDAI and stroke was not statistically significant, whether CDAI was used as a continuous variable or as a four-categorical variable. In patients with BMI ≥25, a statistically significant negative association between BMI and stroke remained.

**Table 3:** Odd ratios and 95% confidence intervals for stroke according to CDAI.

Characteristics	Model 1	Model 2	Model 3
All participants (*n* = 42,042)			
Continuous	0.92 (0.90, 0.93)	0.94 (0.92, 0.95)	0.97 (0.96, 0.99)
CDAI quartile			
Q1 (−7.18, −2.26)	Reference	Reference	Reference
Q2 (−2.26, −0.31)	0.79 (0.70, 0.90)	0.81 (0.71, 0.92)	0.92 (0.80, 1.06)
Q3 (−0.31, 2.15)	0.56 (0.49, 0.64)	0.61 (0.53, 0.71)	0.80 (0.68, 0.93)
Q4 (2.15, 12.80)	0.48 (0.42, 0.56)	0.59 (0.51, 0.69)	0.78 (0.66, 0.92)
*p* for trend	<0.05	<0.05	<0.05
BMI < 25 kg/m^2^ (*n* = 12,223)			
Continuous	0.91 (0.88, 0.94)	0.94 (0.91, 0.97)	0.97 (0.94, 1.01)
CDAI quartile			
Q1 (−7.18, −2.26)	Reference	Reference	Reference
Q2 (−2.26, −0.31)	0.86 (0.66, 1.11)	0.92 (0.70, 1.20)	1.14 (0.85, 1.53)
Q3 (−0.31, 2.15)	0.62 (0.47, 0.83)	0.72 (0.54, 0.96)	0.94 (0.68, 1.30)
Q4 (2.15, 12.80)	0.48 (0.36, 0.65)	0.64 (0.47, 0.86)	0.85 (0.61, 1.19)
*p* for trend	<0.05	<0.05	0.27
BMI ≥ 25 kg/m^2^ (*n* = 29,819)			
Continuous	0.92 (0.90, 0.94)	0.94 (0.92, 0.96)	0.97 (0.95, 1.00)
CDAI quartile			
Q1 (−7.18, −2.26)	Reference	Reference	Reference
Q2 (−2.26, −0.31)	0.77 (0.67, 0.89)	0.77 (0.67, 0.90)	0.86 (0.73, 1.01)
Q3 (−0.31, 2.15)	0.54 (0.46, 0.63)	0.58 (0.49, 0.68)	0.76 (0.63, 0.91)
Q4 (2.15, 12.80)	0.49 (0.41, 0.57)	0.58 (0.49, 0.69)	0.76 (0.63, 0.92)
*p* for trend	<0.05	<0.05	<0.05

Model 1: Non-adjusted.

Model 2: Adjusted for age, and sex.

Model 3: Adjusted for age, sex, education level, PIR, race/ethnicity, smoking, drinking, METs/week, BMI, eGFR, diabetes, hypertension, and hyperlipidemia.

The BMI variables were not adjusted in the stratified analysis of BMI. CDAI, composite dietary antioxidant index; PIR, poverty income ratio; MET, metabolic equivalent of task; BMI, body mass index; eGFR, estimated glomerular filtration rate.

**Figure 3:**
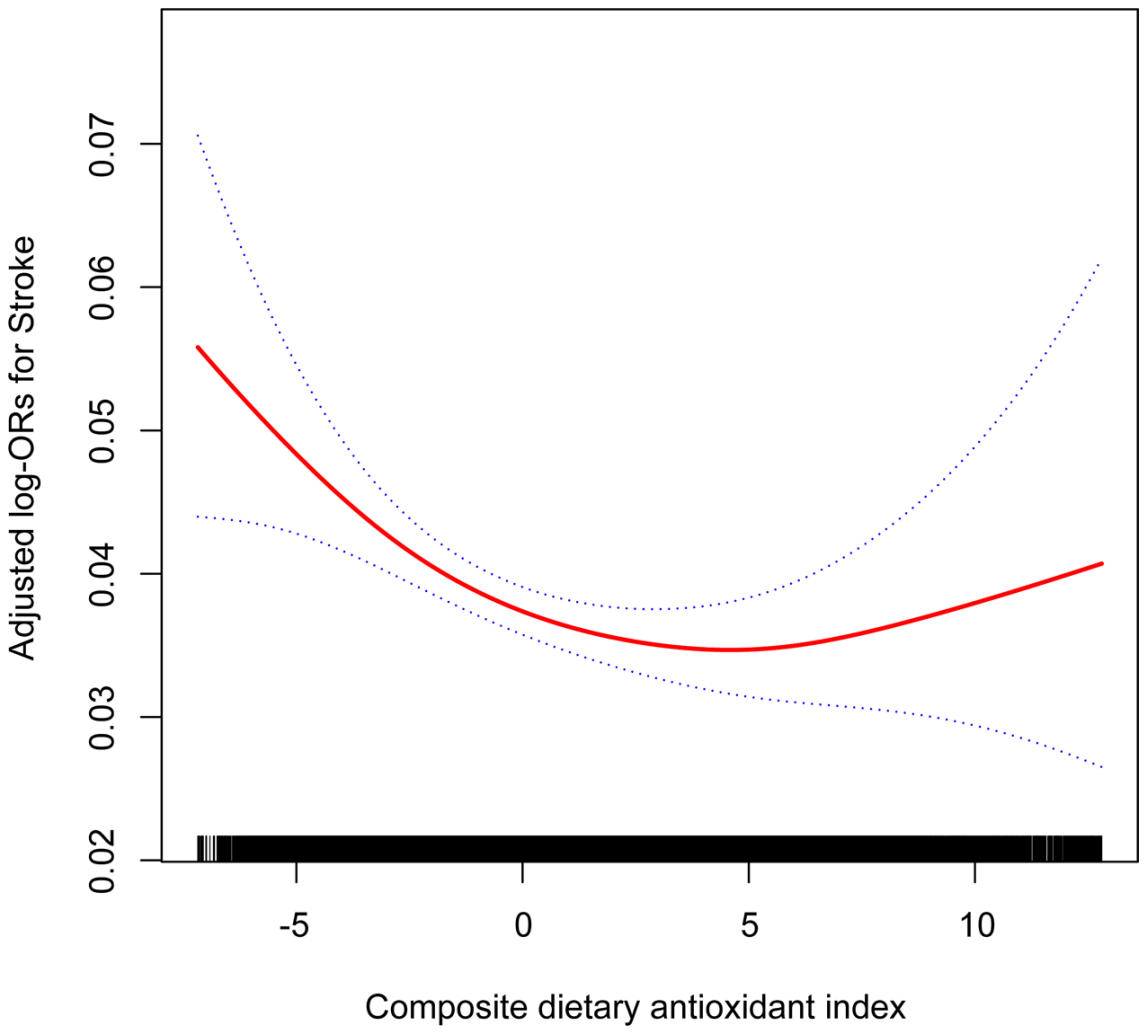
Association between CDAI and the prevalence of stroke. Age, sex, education level, PIR, race/ethnicity, smoking, drinking, METs/week, BMI, eGFR, diabetes, hypertension, and hyperlipidemia were adjusted. OR, odd ratio; CDAI, composite dietary antioxidant index; PIR, poverty income ratio; MET, metabolic equivalent of task; BMI, body mass index; eGFR, estimated glomerular filtration rate.

**Figure 4:**
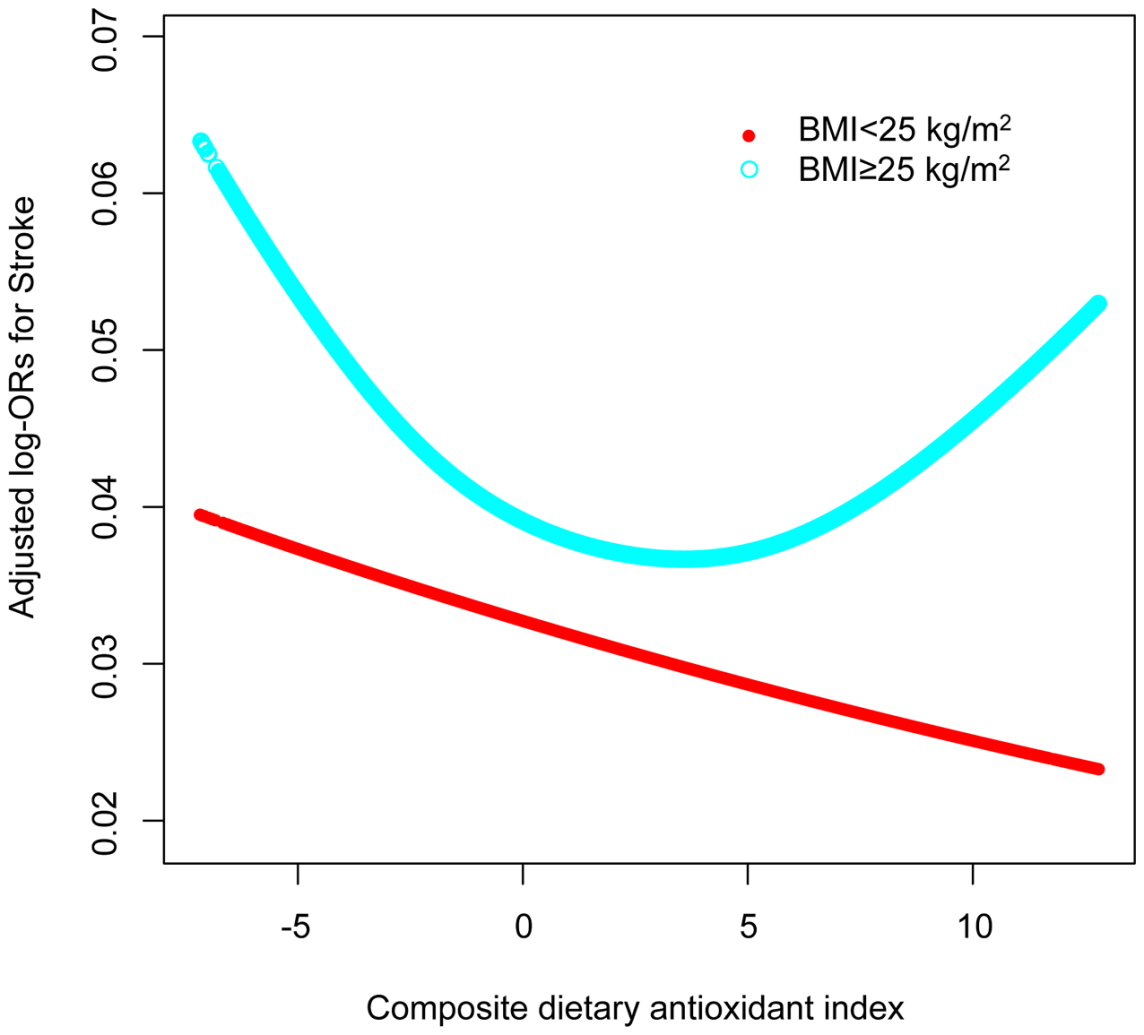
Association between CDAI and the prevalence of stroke among different BMI groups. Age, sex, education level, PIR, race/ethnicity, smoking, drinking, METs/week, BMI, eGFR, diabetes, hypertension, and hyperlipidemia were adjusted. OR, odd ratio; CDAI, composite dietary antioxidant index; BMI, body mass index; PIR, poverty income ratio; MET, metabolic equivalent of task; eGFR, estimated glomerular filtration rate.

GAMs and smooth curve fittings were utilized to assess the relationship between CDAI scores and stroke, revealing a non-linear correlation (adjustments made for age, sex, education level, PIR, race/ethnicity, smoking, drinking, METs/week, BMI, eGFR, diabetes, hypertension, hyperlipidemia) (Figure 3). However, as shown in the analysis in Table 3, there were different relationships between CDAI scores and stroke in different BMI groups. In patients with a BMI <25, there appeared to be a linear negative correlation between CDAI and stroke. However, in patients with a BMI ≥25, the relationship between CDAI and stroke remained non-linear (Figure 4).

For the total population, we identified an inflection point at a CDAI score of 5.95 using two piecewise logistic regression models. To the left of this point, the effect size, 95% confidence interval, and *p* value are 0.96, 0.94–0.98, and 0.0001, respectively. To the right of the inflection point, these values are 1.10, 1.01–1.20, and 0.029, respectively. In patients with BMI < 25, neither the standard linear model nor the two piecewise logistic regression models yielded statistical significance. In patients with BMI ≥ 25, statistical significance was obtained in both standard linear models and two piecewise logistic regression models. After log likelihood ratio test, there was a nonlinear relationship between CDAI and stroke. When CDAI was less than 4.18, there was a negative correlation between CDAI and stroke (OR 0.95, 95% CI 0.92–0.97). When CDAI was greater than 4.18, the relationship between CDAI and stroke was positive (OR 1.09, 95% CI 1.02–1.16) (Table 4).

**Table 4:** Threshold effect analysis of CDAI on stroke using a two-piecewise logistic regression model.

CDAI	Adjusted OR^*^ (95% CI)	*p-*value
All participants (*n* = 42,042)		
Standard linear model	0.97 (0.96, 0.99)	0.0041
Two-piecewise regression model		
Inflection point	5.95	
< Inflection point	0.96 (0.94, 0.98)	0.0001
> Inflection point	1.10 (1.01, 1.20)	0.0290
Log likelihood ratio	/	0.008
BMI < 25 kg/m^2^ (*n* = 12,223)		
Standard linear model	0.97 (0.94, 1.01)	0.1018
Two-piecewise regression model		
Inflection point	−4.39	
< Inflection point	0.74 (0.48, 1.14)	0.1729
> Inflection point	0.98 (0.94, 1.01)	0.2387
Log likelihood ratio	/	0.236
BMI ≥ 25 kg/m^2^ (*n* = 29,819)		
Standard linear model	0.97 (0.95, 1.00)	0.0162
Two-piecewise regression model		
Inflection point	4.18	
< Inflection point	0.95 (0.92, 0.97)	<0.0001
> Inflection point	1.09 (1.02, 1.16)	0.0123
Log likelihood ratio	/	0.001

^*^Adjusted for age, sex, education level, PIR, race/ethnicity, smoking, drinking, METs/week, BMI, eGFR, diabetes, hypertension, and hyperlipidemia. The BMI variables were not adjusted in the stratified analysis of BMI. CDAI, composite dietary antioxidant index; OR, odd ratio; CI, confidence interval; PIR, poverty income ratio; MET, metabolic equivalent of task; BMI, body mass index; eGFR, estimated glomerular filtration rate.

**Figure 5:**
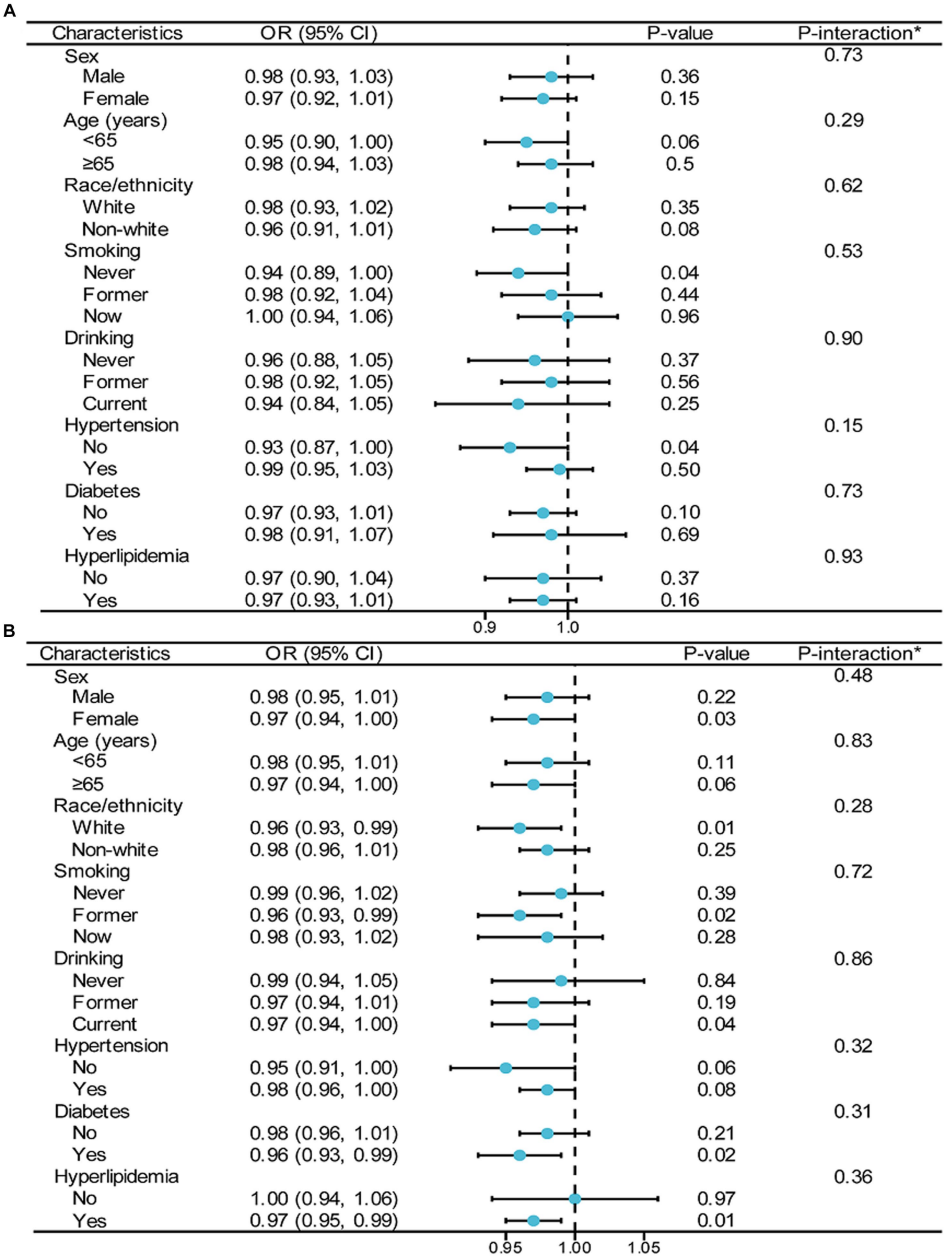
Stratified analyses by potential modifiers of the association between CDAI and the prevalence of stroke among different BMI groups [**(A)** BMI < 25 kg/m^2^; **(B)** BMI ≥ 25 kg/m^2^]. ^*^Each stratification adjusted for all the factors (age, sex, education level, PIR, race/ethnicity, smoking, drinking, METs/week, eGFR, diabetes, hypertension, and hyperlipidemia) except the stratification factor itself. OR, odd ratio; CI, confidence interval; CDAI, composite dietary antioxidant index; BMI, body mass index; PIR, poverty income ratio; MET, metabolic equivalent of task; eGFR, estimated glomerular filtration rate.

To assess the link between CDAI and stroke incidence, a stratified analysis was performed across various BMI categories, examining different subgroups (as depicted in Figure 5). In these BMI groups, factors such as sex, age, race/ethnicity, smoking, drinking, hypertension, diabetes, and hyperlipidemia were found to have no significant impact on the relationship between CDAI scores and stroke prevalence (with all interactions showing *p*-values greater than 0.05).

Finally, the results of partial sample studies that included sleep-related factors as one of the covariates were similar to those described above (Supplementary Tables S1–S3; Supplementary Figures S1, S2).

## 4 Discussion

This research aimed to examine the interaction between CDAI and BMI on stroke risk among U.S. adults. In studies of the general population, we found a significant negative correlation between CDAI scores and stroke incidence, suggesting that higher antioxidant intake is associated with a lower risk of stroke. This association persisted after taking into account variables such as age, sex, education, economic status, race/ethnicity, lifestyle, and comorbidities. However, in further stratified analyses based on BMI, we found that this negative correlation only existed in people with a BMI of ≥25. In addition, nonlinear associations were found in the general population of the United States using GAMs and smooth curve fitting, and this nonlinear association was mainly reflected in the population with a BMI of ≥25. There is a significant inflection point at CDAI = 4.18. The relationship between CDAI and stroke varies on both sides of this point, being negative before this and positive after that. This study is the first to report the interaction between CDAI and BMI on stroke risk in American adults, validates the nonlinear relationship between CDAI and stroke risk, and further stratified the nonlinearity, and provides a comprehensive stratification-based interaction analysis.

Our study observed that stroke incidence escalates with age for both males and females. Supporting this, Bots et al. provided global data indicating that mortality rates from coronary heart disease and stroke ascend with age (24). This pattern is consistent across different countries and genders, underscoring that older individuals have a higher risk of dying from CVD than their younger counterparts. This evidence bolsters the notion that age is a pivotal risk factor for stroke. Moreover, Lasek Bal et al. (25) analyzed stroke risk factors in young patients, highlighting that the influence of certain risk factors, such as hypertension and diabetes, may intensify with age. Similarly, Ananth et al. (26) found that, across different birth cohorts, the mortality rate from ischemic stroke significantly increases with age, further emphasizing age’s critical role in stroke risk. Consequently, age must be considered a key confounding factor in studies investigating CVD like stroke, necessitating its inclusion in further analyses to ensure accurate assessments of other potential risk factors.

Numerous studies have delved into the connection between CDAI and CVD (5, 6, 20, 27, 28). Liu et al. (27) examined this link specifically in postmenopausal women, uncovering a negative association between CDAI levels and the incidence of atherosclerotic cardiovascular disease (ASCVD). They reported that for each standard deviation increase in CDAI, the OR for ASCVD risk is 0.67, with a 95% CI of 0.51–0.88. Zhang et al. (6) extended this research to a broader demographic, concluding that higher CDAI scores, which reflect increased dietary antioxidant intake, are inversely related to a 10-year ASCVD risk in the general population, even after adjusting for potential confounders (OR 0.97, 95% CI 0.95–0.99). This suggests that the inverse correlation between CDAI and ASCVD is applicable across various adult groups. Further, Maugeri et al. (20) focused on the impact of CDAI on carotid intima-media thickness in both men and women, finding that each unit rise in CDAI corresponded to a 4.72 μm reduction in thickness (*p* = 0.018). Wang and Yi’s research expanded the scope to examine CDAI’s influence on overall and cardiovascular mortality. Their findings indicated that a high CDAI is linked to a lower risk of death from all causes and specifically from cardiovascular issues, underscoring the significant role of an antioxidant-rich diet in preventing cardiovascular mortality (5). Additionally, Yang et al. (28) explored this relationship in individuals with type 2 diabetes, discovering that CDAI’s protective effect against CVD mortality is also valid in this subgroup. In conclusion, contemporary research consistently demonstrates a link between higher dietary antioxidant intake, as quantified by CDAI, and a reduced risk of CVD and related mortality. This correlation is evident across diverse groups, including American adults, postmenopausal women, and type 2 diabetes patients. These studies highlight the beneficial impact of antioxidants, suggesting that diets rich in these components could be crucial for cardiovascular health and prevention.

Recent research has highlighted a significant negative correlation between CDAI and stroke risk, suggesting that higher antioxidant intake could reduce stroke incidence (10–13). Mao et al. (12) observed that with each unit increase in CDAI, stroke risk diminished by 4% (OR 0.96, 95% CI 0.93–0.99), and individuals in the highest CDAI quartile had a 37% lower stroke risk compared to those in the lowest quartile (OR 0.63, 95% CI 0.47–0.84). Wang et al. (10) also reported a negative correlation, with their fully adjusted model indicating an OR of 0.97 (95% CI 0.95–0.99) and a 23% decrease in stroke incidence in the highest CDAI quartile versus the lowest (OR 0.77, 95% CI 0.64–0.92). Chen et al. (11) found that each unit increase in CDAI reduced stroke risk by 3.4% (OR 0.966, 95% CI 0.937–0.997), collectively affirming the association between higher dietary antioxidant intake and reduced stroke risk. These findings collectively affirm the link between higher dietary antioxidant intake and reduced stroke risk. Teng et al. (13) also found that higher CDAI scores were associated with a lower stroke risk after adjusting for confounders, with an OR of 0.96 (95% CI 0.94–0.98, *p* < 0.001), and that nomogram models based on antioxidant intake exhibited substantial predictive power for stroke risk, showing an area under the curve (AUC) of 77.4% (76.3–78.5%). Together, these results support the hypothesis that increased dietary antioxidant intake is associated with a diminished risk of stroke, as evidenced by the CDAI.

These studies also explored non-linear relationships between CDAI and stroke risk (10–13). Mao et al. (12) identified a distinct change in this relationship: below a CDAI level of −1.55, each unit increase in CDAI corresponded to a 20% reduction in stroke incidence, but above this level, the correlation ceased. Similarly, Wang et al. (10) found that below a CDAI of −2.99, there was a significant negative correlation with stroke (OR 0.84, 95% CI 0.77–0.93), but this relationship was not significant when CDAI exceeded −2.99 (OR 0.98, 95% CI 0.96–1.01). Chen et al. (11) reported a non-linear association with a turning point at CDAI = 3.078; before this point, each unit increase in CDAI reduced stroke risk by 6.0% (OR 0.940, 95% CI 0.904–0.976), but beyond it, the association was not significant (OR 1.024, 95% CI 0.977–1.074). Teng et al. (13) noted a gradual decline in stroke risk with increasing CDAI until a specific inflection point of 3.66, beyond which the protective effect seemed to stabilize, indicating a potential saturation effect at higher CDAI levels. This is inconsistent with our research findings, although we have also found a non-linear relationship between the two, there are differences in determining the inflection point, which may be due to differences in adjusting variables. While it’s crucial to consider confounders in both linear and non-linear analyses (29), the studies by Wang et al. and Chen et al. may not have adequately accounted for these in their threshold effect analysis (10, 11). If we only consider the curve relationship graph obtained in the study, Wang et al.’s inflection point values in the restricted cubic spline graph might be higher than those in their threshold analysis. Teng et al.’s inflection point is more aligned with our study’s results, so this may be due to their omission in the explanation of confounding factors adjustment. Moreover, the analyses by Teng et al. and Mao et al., though adjusted for confounding factors, likely overlooked additional confounders such as economic status, activity level, renal function, hyperlipidemia, and sleep-related factors (12, 13), all known to impact stroke risk. Economic conditions and physical activity levels have been linked to stroke incidence and mortality (30–33). Renal function, particularly reduced eGFR and chronic kidney disease, is a significant stroke risk factor (34–36). Furthermore, the United States Preventive Service Task Force has identified lipid screening as one of the primary preventive measures for stroke prevention (37), and recent studies have further confirmed the important role of blood lipid levels in the onset and prognosis of stroke (38, 39). Sleep disorders are also acknowledged as a significant risk factor for stroke (40). This gap in confounding factor adjustment might affect the interpretation of CDAI’s impact on stroke risk.

While our current research findings show some alignment with previous studies, they also reveal more intriguing results. Our investigation highlights that the non-linear association between CDAI and stroke incidence primarily manifests in individuals with a BMI of 25 or higher, rather than universally across all populations. Notably, in those with a BMI under 25, the link between CDAI and stroke does not hold statistical significance. This observation aligns with findings from Teng et al. and Mao et al., though their research did not delve into BMI’s role in the CDAI-stroke relationship (12, 13). Considering the differences in variable adjustments mentioned earlier, it becomes essential to undertake more extensive adjustments of variables to further examine the interaction between CDAI and BMI in stroke risk among American adults. Extensive research has established a connection between diet and inflammatory biomarkers, influencing the risk of chronic metabolic diseases either positively or negatively (41–44). The potential preventive impact of healthy dietary patterns, like the Mediterranean diet, is largely attributable to the anti-inflammatory qualities of its key components (45–47). This anti-inflammatory action could mitigate the low-grade inflammation often seen in obese individuals (48, 49). As depicted in Table 1 of our study, individuals in the higher BMI group had notably lower CDAI scores, with stroke incidence escalating in tandem with BMI increases. Thus, BMI may serve as a mediator linking diet, persistent low-grade inflammation, and inflammation-related diseases, going beyond being just a confounding factor. The accumulation of body fat creates an inflammatory metabolic milieu, with BMI showing a positive correlation with inflammatory indicators (50, 51). It is plausible that the interaction mechanism associating CDAI with stroke relates to the link between inflammation and atherosclerosis. Atherosclerosis, a key contributor to CVD including stroke, is acknowledged as an inflammatory condition affecting medium to large blood vessels (52–54). Additionally, inflammation involves immune cell activity and could affect the cardiovascular system through oxidative stress and the formation of foam cells (55, 56). This implies that the dietary influence on inflammation, as indicated by CDAI, could indirectly affect atherosclerosis progression, thereby impacting stroke risk.

However, our study is not without limitations. As an observational study, it is subject to inherent biases and cannot establish causality. The self-reported nature of stroke diagnosis in NHANES could lead to misclassification, and the lack of data on stroke severity or subtype limits the depth of our analysis. These factors necessitate cautious interpretation of our findings and highlight the need for further research to elucidate the underlying biological mechanisms linking dietary antioxidants with stroke risk.

## 5 Conclusion

This study underscores the complex interaction between dietary antioxidant intake and BMI in determining stroke risk among U.S. adults. The findings suggest that individuals with higher BMI may experience more pronounced benefits from dietary antioxidants in stroke prevention. These insights could inform targeted dietary recommendations and public health strategies aimed at reducing stroke risk, particularly in populations with higher BMI. Further research is needed to fully understand these interactions and their implications for stroke prevention guidelines.

While this study elucidates the collective impact of dietary antioxidants on stroke risk among U.S. adults, it does not differentiate the effects of specific antioxidants. Future research should address this gap by investigating which individual antioxidants—such as carotenoids, selenium, vitamin A, vitamin C, vitamin E, and zinc—are most effective at reducing stroke risk, particularly among individuals with higher BMI. This would enable more tailored dietary recommendations and enhance the precision of public health strategies aimed at stroke prevention.

## Data availability statement

The raw data supporting the conclusions of this article will be made available by the authors, without undue reservation.

## Ethics statement

The studies involving humans were approved by National Centre for Health Statistics Institutional Ethics Review Board. The studies were conducted in accordance with the local legislation and institutional requirements. Written informed consent for participation was not required from the participants or the participants' legal guardians/next of kin in accordance with the national legislation and institutional requirements.

## Author contributions

XL: Conceptualization, Data curation, Formal analysis, Investigation, Methodology, Resources, Supervision, Writing – original draft, Writing – review & editing. XH: Conceptualization, Methodology, Resources, Supervision, Validation, Writing – original draft, Writing – review & editing. CY: Funding acquisition, Investigation, Methodology, Resources, Writing – review & editing.
