# Supplementary material for: Interactive effects of Composite Dietary Antioxidant Index with Body Mass Index for the risk of stroke among U.S. adults: insight from NHANES 2001–2018
Source: Front Nutr. 2024 Jun 7;11:1378479. doi: 10.3389/fnut.2024.1378479 (PMC11190190; doi:10.3389/fnut.2024.1378479)
Supplement: Supplementary file 1 [file Data_Sheet_1.DOCX]

**Table S1. Baseline characteristics of subjects (NHANES 2005-2018)**

| **Characteristics** | **BMI < 25 kg/m^2^** | | | | **BMI ≥ 25 kg/m^2^** | | | |
| --- | --- | --- | --- | --- | --- | --- | --- | --- |
|  | **Total**  **n=9590** | **Non-stroke**  **n=9270** | **Stroke**  **n=320** | **P-value** | **Total**  **n=24172** | **Non-stroke**  **n=23138** | **Stroke**  **n=1034** | **P-value** |
| **Age (years)** | 47.06 ± 19.12 | 46.36 ± 18.90 | 67.28 ± 13.95 | <0.001 | 50.92 ± 17.00 | 50.29 ± 16.89 | 65.03 ± 12.83 | <0.001 |
| **Sex, n (%)** |  |  |  | 0.239 |  |  |  | 0.587 |
| Male | 4515 (47.08%) | 4354 (46.97%) | 161 (50.31%) |  | 12169 (50.34%) | 11657 (50.38%) | 512 (49.52%) |  |
| Female | 5075 (52.92%) | 4916 (53.03%) | 159 (49.69%) |  | 12003 (49.66%) | 11481 (49.62%) | 522 (50.48%) |  |
| **Education level, n (%)** |  |  |  | <0.001 |  |  |  | <0.001 |
| Less than high school | 2093 (21.85%) | 1972 (21.30%) | 121 (37.93%) |  | 6212 (25.72%) | 5873 (25.40%) | 339 (32.79%) |  |
| High school | 2100 (21.93%) | 2029 (21.92%) | 71 (22.26%) |  | 5717 (23.67%) | 5416 (23.42%) | 301 (29.11%) |  |
| More than high school | 5384 (56.22%) | 5257 (56.78%) | 127 (39.81%) |  | 12226 (50.61%) | 11832 (51.17%) | 394 (38.10%) |  |
| **PIR, n (%)** |  |  |  | <0.001 |  |  |  | <0.001 |
| Low | 2663 (30.23%) | 2546 (29.92%) | 117 (38.74%) |  | 6941 (31.40%) | 6562 (31.01%) | 379 (40.02%) |  |
| Medium | 3272 (37.14%) | 3146 (36.98%) | 126 (41.72%) |  | 8550 (38.67%) | 8140 (38.47%) | 410 (43.29%) |  |
| High | 2875 (32.63%) | 2816 (33.10%) | 59 (19.54%) |  | 6617 (29.93%) | 6459 (30.52%) | 158 (16.68%) |  |
| **Race/ethnicity, n (%)** |  |  |  | <0.001 |  |  |  | <0.001 |
| Non-Hispanic White | 4396 (45.84%) | 4231 (45.64%) | 165 (51.56%) |  | 10058 (41.61%) | 9553 (41.29%) | 505 (48.84%) |  |
| Non-Hispanic Black | 1750 (18.25%) | 1671 (18.03%) | 79 (24.69%) |  | 5535 (22.90%) | 5240 (22.65%) | 295 (28.53%) |  |
| Mexican American | 969 (10.10%) | 944 (10.18%) | 25 (7.81%) |  | 4287 (17.74%) | 4183 (18.08%) | 104 (10.06%) |  |
| Others | 2475 (25.81%) | 2424 (26.15%) | 51 (15.94%) |  | 4292 (17.76%) | 4162 (17.99%) | 130 (12.57%) |  |
| **Smoking, n (%)** |  |  |  | <0.001 |  |  |  | <0.001 |
| Never | 5263 (54.88%) | 5152 (55.58%) | 111 (34.69%) |  | 13305 (55.04%) | 12896 (55.74%) | 409 (39.56%) |  |
| Former | 1825 (19.03%) | 1723 (18.59%) | 102 (31.87%) |  | 6374 (26.37%) | 5975 (25.82%) | 399 (38.59%) |  |
| Now | 2499 (26.06%) | 2392 (25.80%) | 107 (33.44%) |  | 4481 (18.54%) | 4255 (18.39%) | 226 (21.86%) |  |
| Not reported | 3 (0.03%) | 3 (0.03%) | 0 (0.00%) |  | 12 (0.05%) | 12 (0.05%) | 0 (0.00%) |  |
| **Drinking, n (%)** |  |  |  | <0.001 |  |  |  | <0.001 |
| Never | 1235 (12.88%) | 1186 (12.79%) | 49 (15.31%) |  | 3102 (12.83%) | 2964 (12.81%) | 138 (13.35%) |  |
| Former | 1189 (12.40%) | 1103 (11.90%) | 86 (26.88%) |  | 3906 (16.16%) | 3578 (15.46%) | 328 (31.72%) |  |
| Mild | 3034 (31.64%) | 2945 (31.77%) | 89 (27.81%) |  | 7384 (30.55%) | 7108 (30.72%) | 276 (26.69%) |  |
| Moderate | 1459 (15.21%) | 1432 (15.45%) | 27 (8.44%) |  | 3326 (13.76%) | 3241 (14.01%) | 85 (8.22%) |  |
| Heavy | 1811 (18.88%) | 1784 (19.24%) | 27 (8.44%) |  | 4438 (18.36%) | 4338 (18.75%) | 100 (9.67%) |  |
| Not reported | 862 (8.99%) | 820 (8.85%) | 42 (13.12%) |  | 2016 (8.34%) | 1909 (8.25%) | 107 (10.35%) |  |
| **METs/week, n (%)** |  |  |  | <0.001 |  |  |  | <0.001 |
| Low | 1649 (17.19%) | 1597 (17.23%) | 52 (16.25%) |  | 4252 (17.59%) | 4097 (17.71%) | 155 (14.99%) |  |
| Moderate | 201 (2.10%) | 197 (2.13%) | 4 (1.25%) |  | 425 (1.76%) | 416 (1.80%) | 9 (0.87%) |  |
| Vigorous | 5614 (58.54%) | 5488 (59.20%) | 126 (39.38%) |  | 12765 (52.81%) | 12400 (53.59%) | 365 (35.30%) |  |
| Not reported | 2126 (22.17%) | 1988 (21.45%) | 138 (43.12%) |  | 6730 (27.84%) | 6225 (26.90%) | 505 (48.84%) |  |
| **BMI (kg/m^2^)** | 22.18 ± 2.02 | 22.17 ± 2.01 | 22.29 ± 2.13 | 0.319 | 32.05 ± 6.19 | 32.04 ± 6.20 | 32.30 ± 5.84 | 0.182 |
| **SBP (mmHg)** | 120.83 ± 19.52 | 120.37 ± 19.16 | 134.14 ± 24.65 | <0.001 | 125.38 ± 18.05 | 125.06 ± 17.80 | 132.59 ± 21.74 | <0.001 |
| **DBP (mmHg)** | 68.82 ± 11.40 | 68.81 ± 11.31 | 69.07 ± 13.86 | 0.692 | 71.20 ± 12.13 | 71.31 ± 12.05 | 68.77 ± 13.52 | <0.001 |
| **eGFR (ml/min/1.73 m^2^)** | 96.89 ± 23.59 | 97.69 ± 23.07 | 73.46 ± 26.66 | <0.001 | 92.11 ± 23.64 | 93.01 ± 23.16 | 71.63 ± 24.91 | <0.001 |
| **Diabetes, n (%)** | 858 (8.95%) | 777 (8.38%) | 81 (25.31%) | <0.001 | 5505 (22.77%) | 5018 (21.69%) | 487 (47.10%) | <0.001 |
| **Hypertension, n (%)** | 2796 (29.16%) | 2561 (27.63%) | 235 (73.44%) | <0.001 | 11720 (48.49%) | 10854 (46.91%) | 866 (83.75%) | <0.001 |
| **Hyperlipidemia, n (%)** | 5152 (53.73%) | 4910 (52.97%) | 242 (75.62%) | <0.001 | 18297 (75.70%) | 17396 (75.19%) | 901 (87.14%) | <0.001 |
| **Trouble sleeping, n (%)** | 2059 (21.48%) | 1951 (21.05%) | 108 (33.86%) | <0.001 | 6621 (27.40%) | 6150 (26.59%) | 471 (45.55%) | <0.001 |
| **CDAI** | 0.43 ± 3.65 | 0.47 ± 3.65 | -0.51 ± 3.46 | <0.001 | 0.20 ± 3.50 | 0.24 ± 3.49 | -0.66 ± 3.44 | <0.001 |

**Abbreviations:** PIR, poverty income ratio; MET, metabolic equivalent of task; BMI, body mass index; SBP, systolic blood pressure; DBP, diastolic blood pressure; eGFR, estimated glomerular filtration rate; CDAI, composite dietary antioxidant index.

**Table S2. Odd ratios and 95% confidence intervals for stroke according to CDAI (NHANES 2005-2018)**

| **Characteristics** | **Model 1** | **Model 2** | **Model 3** |
| --- | --- | --- | --- |
| **All participants (n=33762)** | | | |
| **Continuous** | 0.92 (0.90, 0.94) | 0.94 (0.92, 0.96) | 0.97 (0.96, 0.99) |
| **CDAI Quartile** |  |  |  |
| Q1 (-7.18, -2.26) | Reference | Reference | Reference |
| Q2 (-2.26, -0.31) | 0.74 (0.64, 0.85) | 0.75 (0.65, 0.86) | 0.84 (0.72, 0.99) |
| Q3 (-0.31, 2.15) | 0.56 (0.48, 0.65) | 0.60 (0.51, 0.70) | 0.78 (0.66, 0.93) |
| Q4 (2.15, 12.80) | 0.48 (0.41, 0.56) | 0.58 (0.49, 0.68) | 0.75 (0.62, 0.89) |
| **P for trend** | <0.05 | <0.05 | <0.05 |
| **BMI < 25 kg/m^2^ (n=9590)** | | | |
| **Continuous** | 0.92 (0.89, 0.95) | 0.94 (0.91, 0.98) | 0.97 (0.94, 1.01) |
| **CDAI Quartile** |  |  |  |
| Q1 (-7.18, -2.26) | Reference | Reference | Reference |
| Q2 (-2.26, -0.31) | 0.75 (0.56, 1.01) | 0.79 (0.59, 1.07) | 1.02 (0.73, 1.42) |
| Q3 (-0.31, 2.15) | 0.58 (0.42, 0.79) | 0.64 (0.46, 0.88) | 0.87 (0.61, 1.25) |
| Q4 (2.15, 12.80) | 0.53 (0.39, 0.72) | 0.67 (0.49, 0.92) | 0.88 (0.61, 1.26) |
| **P for trend** | <0.05 | <0.05 | 0.37 |
| **BMI ≥ 25 kg/m^2^ (n=24172)** | | | |
| **Continuous** | 0.92 (0.90, 0.94) | 0.94 (0.92, 0.96) | 0.97 (0.95, 0.99) |
| **CDAI Quartile** |  |  |  |
| Q1 (-7.18, -2.26) | Reference | Reference | Reference |
| Q2 (-2.26, -0.31) | 0.73 (0.62, 0.86) | 0.73 (0.62, 0.86) | 0.79 (0.66, 0.95) |
| Q3 (-0.31, 2.15) | 0.55 (0.46, 0.65) | 0.59 (0.49, 0.70) | 0.76 (0.62, 0.92) |
| Q4 (2.15, 12.80) | 0.47 (0.39, 0.57) | 0.56 (0.46, 0.67) | 0.70 (0.57, 0.86) |
| **P for trend** | <0.05 | <0.05 | <0.05 |

**Model 1:** Non-adjusted.

**Model 2:** Adjusted for age, and sex.

**Model 3:** Adjusted for age, sex, education level, PIR, race/ethnicity, smoking, drinking, METs/week, BMI, eGFR, diabetes, hypertension, hyperlipidemia, and trouble sleeping.

**Notes:** The BMI variables were not adjusted in the stratified analysis of BMI.

**Abbreviations:** CDAI, composite dietary antioxidant index; PIR, poverty income ratio; MET, metabolic equivalent of task; BMI, body mass index; eGFR, estimated glomerular filtration rate.

**Table S3.** **Threshold effect analysis of CDAI on stroke using a two-piecewise logistic regression model (NHANES 2005-2018)**

| **CDAI** | **Adjusted OR^*^ (95% CI)** | **P-value** |
| --- | --- | --- |
| **All participants (n=33762)** | | |
| **Standard linear model** | 0.97 (0.96, 0.99) | 0.0060 |
| **Two-piecewise regression model** |  |  |
| Inflection point | 6.30 |  |
| < Inflection point | 0.96 (0.94, 0.98) | 0.0002 |
| > Inflection point | 1.11 (1.01, 1.22) | 0.0292 |
| **Log likelihood ratio** | / | 0.009 |
| **BMI < 25 kg/m^2^ (n=9590)** | | |
| **Standard linear model** | 0.97 (0.94, 1.01) | 0.1692 |
| **Two-piecewise regression model** |  |  |
| Inflection point | -1.29 |  |
| < Inflection point | 0.97 (0.93, 1.02) | 0.2319 |
| > Inflection point | 0.98 (0.79, 1.21) | 0.8517 |
| **Log likelihood ratio** | / | 0.9532 |
| **BMI ≥ 25 kg/m^2^ (n=24172)** | | |
| **Standard linear model** | 0.97 (0.95, 0.99) | 0.0141 |
| **Two-piecewise regression model** |  |  |
| Inflection point | 6.30 |  |
| < Inflection point | 0.95 (0.93, 0.98) | 0.0003 |
| > Inflection point | 1.15 (1.04, 1.28) | 0.0084 |
| **Log likelihood ratio** | / | 0.003 |

**Notes:** ^*^Adjusted for age, sex, education level, PIR, race/ethnicity, smoking, drinking, METs/week, BMI, eGFR, diabetes, hypertension, hyperlipidemia, and trouble sleeping. The BMI variables were not adjusted in the stratified analysis of BMI.

**Abbreviations:** CDAI, composite dietary antioxidant index; OR, odd ratio; CI, confidence interval; PIR, poverty income ratio; MET, metabolic equivalent of task; BMI, body mass index; eGFR, estimated glomerular filtration rate.


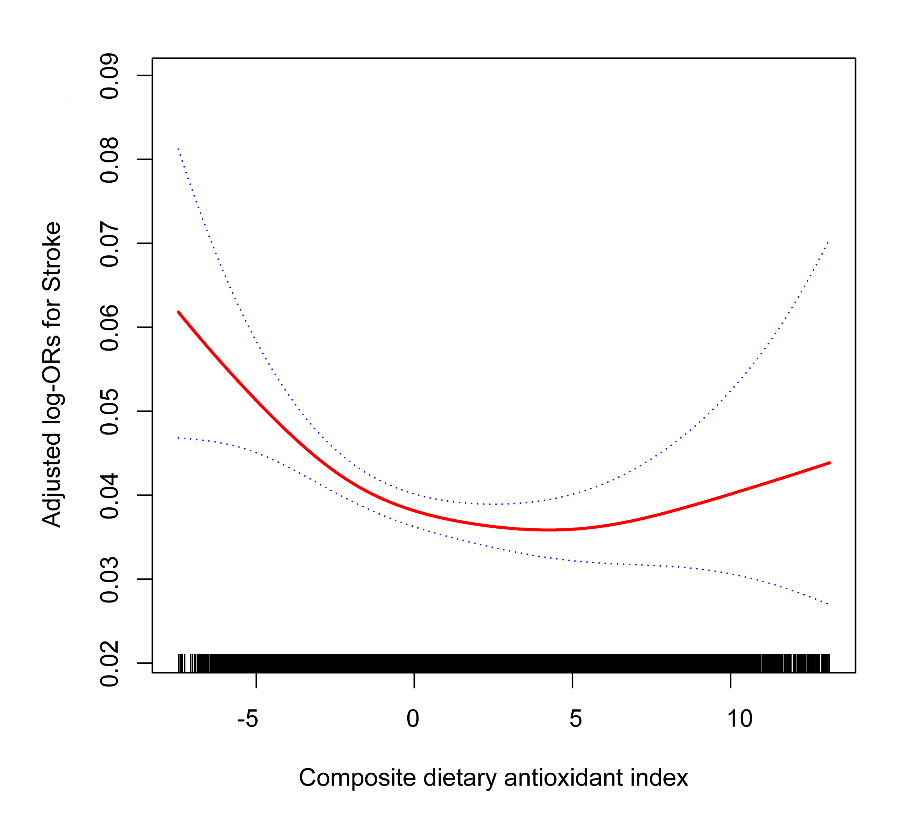


**Figure S1. Association between CDAI and the prevalence of stroke (NHANES 2005-2018)**

**Notes:** Age, sex, education level, PIR, race/ethnicity, smoking, drinking, METs/week, BMI, eGFR, diabetes, hypertension, hyperlipidemia, and trouble sleeping were adjusted.

**Abbreviations:** OR, odd ratio; CDAI, composite dietary antioxidant index; PIR, poverty income ratio; MET, metabolic equivalent of task; BMI, body mass index; eGFR, estimated glomerular filtration rate.


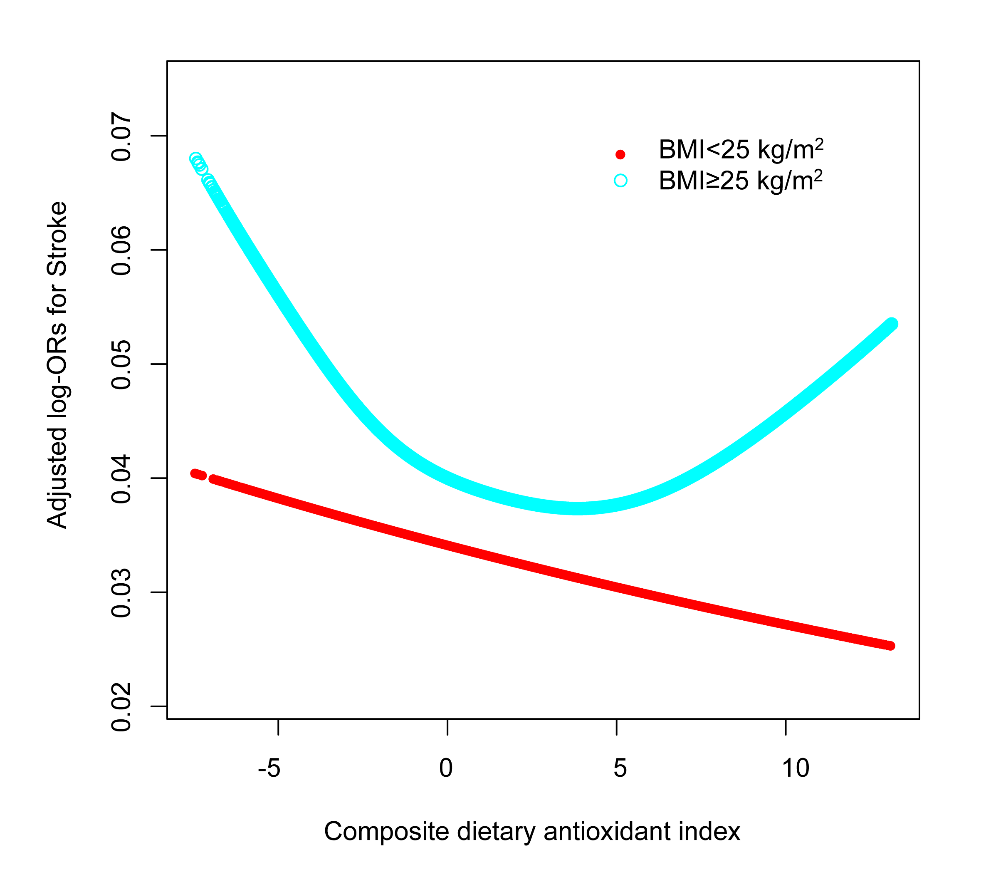


**Figure S2. Association between CDAI and the prevalence of stroke among different BMI groups (NHANES 2005-2018)**

**Notes:** Age, sex, education level, PIR, race/ethnicity, smoking, drinking, METs/week, BMI, eGFR, diabetes, hypertension, hyperlipidemia, and trouble sleeping were adjusted.

**Abbreviations:** OR, odd ratio; CDAI, composite dietary antioxidant index; BMI, body mass index; PIR, poverty income ratio; MET, metabolic equivalent of task; eGFR, estimated glomerular filtration rate.
